# Autism spectrum disorder: overdiagnosis or a new pandemic?

**DOI:** 10.1016/j.jped.2025.101439

**Published:** 2025-09-22

**Authors:** Kamila Castro, Rudimar Riesgo, Carlos Gadia

**Affiliations:** a Child Neurology Unit, Pediatrics Service, Hospital de Clínicas de Porto Alegre (HCPA), Porto Alegre, RS, Brazil; b Translational Research Group in Autism Spectrum Disorders (GETTEA), Universidade Federal Do Rio Grande Do Sul (UFRGS), Porto Alegre, RS, Brazil; c Pediatric Neurologist - Nicklaus Children's Hospital, Miami, USA

**Keywords:** Autism spectrum disorder, Epidemiology, Prevalence, Overdiagnosis, Diagnostic criteria

## Abstract

**Objective:**

To critically analyze the factors influencing prevalence estimates of Autism Spectrum Disorder (ASD), considering methodological, clinical, etiological, and sociocultural determinants that shape epidemiological data and diagnostic practices.

**Data synthesis:**

In recent decades, a substantial increase in ASD prevalence has been observed globally. This phenomenon is shaped by a combination of factors, including changes in diagnostic criteria, improved detection methods, expanded access to health services, and greater public awareness. However, it also raises concerns about possible overdiagnosis, particularly in complex clinical contexts. The interpretation of prevalence data is influenced by methodological designs, population characteristics, and sociocultural dynamics.

**Summary of the findings:**

The absence of biological markers, the high rate of psychiatric comorbidities, and disparities in access to qualified professionals further complicate the diagnostic process. These elements highlight the need for caution when comparing data across studies, time periods, or geographic regions.

**Conclusion:**

The ASD prevalence reflects a multifaceted process that demands careful and comprehensive interpretation. A deeper understanding of this scenario requires critical reflection on how diagnoses are established, interpreted, and applied. Strengthening diagnostic practices and epidemiological approaches is essential for ensure more accurate data and support informed decision-making in health policies.

## Introduction

Historically, numbers have played a decisive role in shaping clinical strategies related to the health-disease process. Some of this data comes from epidemiological studies. Epidemiology is the science that studies the distribution and determinants of health-related states or events in specific populations, and the application of this knowledge to the control of health problems. In other words, epidemiology investigates how, when, where, and why diseases occur in groups of people, with the aim of preventing, controlling, and improving public health. It employs statistical and analytical methods to identify patterns, risk factors, and the impact of interventions, playing a vital role in the development of health policies, in service planning, and in evaluation of prevention programs. Epidemiologists use various measures, such as prevalence (the proportion of existing cases in a population at a given time), incidence (the number of new cases during a specific period), and cumulative incidence (the proportion of individuals who develop the condition over a defined period) [1].

Several studies have reported an increase in the prevalence of Autism Spectrum Disorder (ASD) over the past decades, sparking intense debates about the possibility of overdiagnosis [2]. While the expansion of diagnosis may reflect advances in early identification and the inclusion of previously overlooked subgroups, it also raises concerns about excessive diagnoses, especially in borderline cases. By definition, overdiagnosis does not necessarily mean that someone has been “wrongly” diagnosed. It means that in some cases, the diagnosis may be applied more broadly than necessary, or that behaviors previously considered within the typical range of development are now being labeled as pathological. Discrepancies in these numbers raise important questions that must be discussed to achieve a better understanding of the diagnostic process and, consequently, of the cascade of actions that follow its confirmation or exclusion.

This article proposes a critical analysis of the multiple factors that may influence ASD data, assessing the robustness and consistency of the available evidence, as well as the risks associated with diagnostic overestimation or underestimation.

## A historical perspective on autism epidemiology

The first epidemiological data on autism were described in 1966 by Victor Lotter [3]. Approximately 76,000 children were evaluated in the United Kingdom, and the reported autism prevalence was 4.5 cases per 10,000 children. Lotter not only provided an initial quantitative reference but also marked a profound reconfiguration of the concept of autism. His work reflected a paradigmatic shift in British child psychiatry: the move away from psychoanalytic explanations based on fantasy and unconscious symbolism toward observational and behavioral criteria, which were more compatible with the epidemiological approach gaining traction after the 1959 Mental Health Act [4]. By proposing a set of measurable behavioral traits for autism, Lotter made large-scale statistical investigation possible, establishing a new diagnostic logic. This study was decisive not only for the consolidation of autism as a distinct nosological category but also for its incorporation into public mental health policies at a time of psychiatric deinstitutionalization and the expansion of community-based services in the United Kingdom [5].

Over time, numerous studies have reported data of varying magnitudes from different parts of the world. Globally, estimates suggest that approximately 1 % of the world population is on the autism spectrum, although this number varies depending on the region and the methodology used [6]. A meta-analysis based on 74 studies involving more than 30 million participants estimated the global prevalence of autism at 0.6 % (95 % CI: 0.4–1 %), with regional variations of 0.4 % in Asia, 1 % in the Americas and Africa, 0.5 % in Europe, and up to 1.7 % in Australia [7].

Even in the United States—a country with extensive health surveillance—autism prevalence estimates vary significantly across different agencies and regions, reflecting differences in data collection methodologies and diagnostic criteria. According to the most recent data from the Centers for Disease Control and Prevention (CDC), through the Autism and Developmental Disabilities Monitoring (ADDM) Network, the prevalence of autism among 8-year-old children in 2022 was 1 in 31 (3.2 %). However, this rate varied widely across the 16 monitored sites, ranging from 0.97 % in Laredo, Texas, to 5.31 % in California [8]. The National Health Interview Survey (NHIS), which is based on household interviews with parents, estimated a prevalence of 3.05 % among children aged 3 to 17 years during the period from 2019 to 2021 [9]. This approach, by relying on self-reports, may capture diagnoses that are not documented in health or education systems.

In Brazil, preliminary data were presented for the first time, revealing that 2.4 million people reported having received an ASD diagnosis from a healthcare professional, representing 1.2 % of the national population. Prevalence was higher among children and adolescents, especially in the 5 to 9 age group, where 2.6 % had been diagnosed, with a notable 3.8 % prevalence among boys in this age range. Additionally, a higher proportion of diagnoses was observed among individuals who self-identified as white compared to other ethnic-racial groups [10].

## Determinants of epidemiological data in autism spectrum disorder

The generation of epidemiological data on ASD is influenced by a combination of different factors. In this context, a critical analysis of the determinants of such data is essential for the accurate interpretation of prevalence rates, helping to avoid both overestimations and significant omissions. These factors range from issues related to foundational research to the dissemination of information and the public portrayal of autism.

## Methodological factors

Methodological factors play a central role in the generation of epidemiological data on ASD, as they often encompass and interconnect other determining elements such as social, demographic, and technical aspects. In many cases, methodological choices—such as study design, adopted diagnostic criteria, data collection instruments, and the definition of target populations — reflect and incorporate these broader dimensions. Thus, factors like increased access to services, greater awareness of the disorder, or even the way data are publicly disseminated may be embedded within methodological decisions and should not be analyzed in isolation. Recognizing this overlap is essential to understanding the complexity involved in interpreting the data.

Furthermore, according to Fombonne (2019) [11, 12, 13], three essential components related to study methodology can shape the results: case definition, case identification, and case evaluation. Table 1 presents these concepts along with examples that can be applied in autism research.

**Table 1:** Case definition, identification, and evaluation: key methodological components in autism spectrum disorder research*.

Element	Description	Example in Autism Studies
Case Definition	It refers to the set of standardized criteria used to identify and classify an individual as having (or not having) a particular health condition. These criteria may include clinical signs, symptoms, laboratory results, epidemiological data, and exposure factors. In the context of epidemiological surveillance and scientific research, the case definition is essential to ensure consistency, comparability, and validity of the collected data. The case definition determines who will be included in a study (e.g., cases and controls).	It is the standardized set of clinical criteria used to classify an individual as a case of ASD (or not). These criteria should be consistent with diagnostic manuals such as the DSM-5-TR or ICD-11.
Case Identification	It refers to the process of detecting, recognizing, and recording individuals who meet the criteria established in a case definition for a given health condition. Different strategies can be designed and applied to identify individuals who meet the study’s inclusion criteria. For example, some countries have population-based databases, which can serve as sources of information for epidemiological studies. However, these also carry the bias that only the portion of the population registered in such databases will be analyzed. Another strategy, for instance, may involve combining data from multiple collection sites.	How individuals with ASD are located and selected to compose the study sample. For example, studies may extract information from population-based databases or use multicenter approaches (treatment centers, schools, and hospitals).
Case Evaluation	It refers to the set of procedures used to verify whether an individual meets the criteria defined in the case definition for a given health condition. The use of validated scales, checklists, and tests contributes to this evaluation process.	In ASD, this involves a multimodal approach: structured clinical interviews, review of medical and family history, direct behavioral observation, and the use of standardized instruments such as the ADOS-2 and the ADI-R. The absence of biological markers makes methodological standardization and evaluator training essential to ensure diagnostic reliability.

*ASD, Autism Spectrum Disorder; DSM-5-TR, Diagnostic and Statistical Manual of Mental Disorders, Fifth Edition, Text Revision; ICD-11, International Classification of Diseases 11th Revision; ADOS-2, Autism Diagnostic Observation Schedule- 2; ADI-R, Autism Diagnostic Interview.

### Demographic factors

Demographic differences across epidemiological studies represent a significant variable in the interpretation of prevalence rates. Factors such as age, sex, race, socioeconomic status, and the geographic location of the studied populations directly influence the results obtained. Many studies focus on urban regions with greater access to healthcare and diagnostic services, which can lead to the underrepresentation of rural or socially vulnerable populations. Research has shown higher prevalence rates among families with higher income and education levels, likely due to better access to diagnostic services [14]. Moreover, the predominance of samples composed of certain ethnic groups or age ranges can limit the generalizability of findings to broader contexts. When such demographic disparities are not controlled for or clearly reported, they compromise the comparability between studies and may contribute to an unequal understanding of autism across different population segments.

It is also important to note that, in many countries, a formal diagnosis serves as a criterion for eligibility for public services, creating institutional and familial incentives to obtain official diagnostic reports [15]. This dynamic may contribute to increased diagnostic rates and lends support to the hypothesis of potential overdiagnosis.

### Clinical and etiological factors

The expansion of diagnostic criteria for autism is widely recognized as a major contributing factor to the increase in prevalence data. The concept of autism has undergone profound reformulations throughout the 20th century, shifting from being viewed as a symptom of schizophrenia to becoming an autonomous diagnostic category within the field of neurodevelopmental disorders.

While in the 1940s and 1950s autism was associated with excessive fantasy, beginning in the 1960s, researchers such as Michael Rutter started to describe it as a condition marked by observable deficits in behavior, language, and social interaction. This shift culminated in the development of more objective and replicable diagnostic criteria, which were further strengthened with the publication of the DSM-III (1980), and more recently, the DSM-5 (2013), which consolidated the concept of a spectrum and eliminated categories such as “Asperger’s syndrome” and “childhood autism.” This trajectory reflects not only scientific advances but also paradigm shifts in how childhood development, normality, and neurological diversity are understood.

The inclusion or exclusion of comorbidities—such as learning disabilities, attention-deficit/hyperactivity disorder (ADHD), language disorders, or intellectual disability—directly influences the eligibility criteria used in epidemiological studies and, consequently, the reported prevalence rates. Research shows that ASD rarely occurs in isolation. According to Lai et al. (2014) [16], about 70 % of individuals with ASD present at least one psychiatric comorbidity, and approximately 40 % present two or more.

This high rate of co-occurrence makes diagnosis more complex, especially when comorbid symptoms overlap with the core features of autism. Such overlap may lead to misdiagnoses, overdiagnosis, or even underreporting of atypical cases. Furthermore, the presence of comorbidities can alter the way core ASD symptoms manifest, complicating the uniform application of diagnostic criteria across different populations [17,18]. The decision to include or exclude individuals with other neurodevelopmental conditions can artificially inflate or reduce prevalence rates, hindering comparisons across contexts and over time.

A recent study aimed to verify ASD diagnoses in children aged 5–12 through a standardized clinical reassessment conducted by specialists. All participants had previously received an ASD diagnosis outside of a research setting and were re-evaluated using the ADI-R, ADOS-2, and behavioral questionnaires completed by parents and teachers. In total, 53 % (122 children) had their ASD diagnosis confirmed by the evaluators, while 47 % (110 children) did not meet the formal diagnostic criteria for ASD. The study found that children who did not meet the criteria were more likely to present with other disorders, such as anxiety disorders, attention deficits, and language impairments. These findings suggest potential diagnostic errors, particularly in cases involving psychiatric comorbidities or nonspecific atypical development. The authors recommend more careful and multidisciplinary diagnostic evaluations, especially in research settings and public policy contexts [19].

Genetic components play a central role in the etiology of ASD and are widely recognized as one of the main risk factors for the development of the condition [20]. Genomic research has identified hundreds of genetic variants associated with autism, including de novo mutations, rare high-impact variants, and common polymorphisms that collectively contribute to susceptibility to the disorder [21]. Despite this, no single mutation accounts for the majority of cases, reinforcing the notion of a complex polygenic basis, with multiple genes interacting with each other and with environmental factors. Through their interaction with genetic factors, environmental factors also form part of the set of variables that influence the clinical aspects studied in autism. These factors do not cause ASD in isolation but may disrupt sensitive biological processes during fetal neurodevelopment, amplifying underlying genetic vulnerabilities. The heterogeneity of findings suggests that environmental effects are, in many cases, modulated by epigenetic mechanisms or gene–environment interactions, making their investigation particularly complex [22,23]. This etiological complexity — characterized by the diversity of genetic and environmental mechanisms — has a direct impact on epidemiological studies, often hindering the standardization of diagnostic criteria and the comparison between populations. Therefore, attributing the increase in autism prevalence solely to environmental causes constitutes an inadequate oversimplification. A more accurate understanding of these numbers requires an integrated approach that considers both advances in genetics and the complementary influence of environmental factors on the development of the disorder.

### Advances in technology and science

Scientific and technological advances in recent decades have played a central role in the diagnostic reconfiguration of autism, directly impacting prevalence data. The rise in ASD statistics should not be interpreted solely as a reflection of a biological epidemic, but rather as an expression of scientific transformations in how “autism” is currently understood. Recent research in neuroscience has significantly deepened our understanding of the biological underpinnings of ASD. Neuroimaging studies — such as structural and functional magnetic resonance imaging — have identified atypical patterns of brain connectivity, alterations in regions such as the prefrontal cortex, amygdala, and corpus callosum, as well as differences in synaptic development in children with autism [24,25]. These findings have reinforced the notion that autism has a measurable biological basis, even though such evidence has not yet been fully incorporated into clinical practice through specific diagnostic biomarkers.

The search for both genetic and neurofunctional biomarkers has driven the development of increasingly early screening programs, enabling diagnoses before the age of three. The widespread use of standardized instruments such as the Autism Diagnostic Observation Schedule – Second Edition (ADOS-2) and the Autism Diagnostic Interview – Revised (ADI-R) has promoted greater systematization and comparability across clinical evaluations and multicenter epidemiological studies, reducing subjectivity in the diagnostic process [26, 27].

Recently, an innovative technology — Earlipoint® — was developed to detect signs of autism in children between 16 and 30 months of age. The technology uses an approach known as Dynamic Quantification of Social-Visual Engagement (DQSVE), in which short videos featuring social scenes are shown to children while an eye-tracking system captures their visual behavior. Visual fixation data are analyzed by artificial intelligence algorithms, which generate an objective ASD diagnosis along with three severity indices in the following domains: social impairment (corresponding to the ADOS‑2), verbal ability, and nonverbal ability (corresponding to Mullen Scales scores). This technology represents the first diagnostic biomarker for autism [28, 29].

In addition, interactive platforms such as the Global Autism Prevalence Map, developed by researchers including Eric Fombonne and hosted by the scientific network The Transmitter, have contributed to real-time data visualization, helping to identify regional, methodological, and population-level gaps in global prevalence studies.

Thus, scientific advances have not only expanded the understanding of the autism spectrum in neurobiological and behavioral terms, but have also indirectly contributed to the increase in prevalence rates by making diagnostic criteria more sensitive, broadening the age range for diagnosis, and improving population screening methods.

### Sociocultural factors

In recent decades, growing public awareness of autism—driven by advocacy campaigns and the engagement of public figures — has expanded the social and political discourse surrounding the diagnosis. This process has contributed to a reduction in stigma and increased visibility of the topic, which, in turn, has encouraged more people to seek diagnostic evaluations, including adults who spent much of their lives without formal identification.

The widespread dissemination of information about autism, particularly through social media, has played a central role in this landscape. However, this increased access to information has also had significant implications for epidemiological data, especially regarding self-diagnosis. In this context, a phenomenon linked to infodemic — the overabundance of information, often inaccurate or unverified, that circulates widely and confuses the public —has emerged. Many individuals report identifying with descriptions of ASD-related symptoms and behaviors without undergoing specialized clinical assessment or receiving a formal diagnosis. When such self-reports are included in population surveys or self-declared studies, they may influence prevalence estimates and contribute to a possible scenario of overdiagnosis.

Alongside public awareness, there has also been progress in the training of healthcare professionals, with a greater availability of specialists qualified to perform ASD diagnoses. However, this expansion is not always accompanied by adequate training. The lack of specific preparation or the improper use of diagnostic criteria can result in the inaccurate application of clinical classifications, directly affecting data reliability and contributing to misdiagnoses.

Thus, while the growing awareness of ASD represents a significant advancement, it also requires critical attention to the quality of diagnostic processes and how such information is incorporated into statistics and public policies.

## Epidemiological dynamics and contextual factors

Based on the factors mentioned above, it becomes clear that understanding epidemiological data related to autism requires in-depth analyses across multiple domains. Integrating scientific, clinical, demographic, and social aspects is a critical challenge that must be addressed. Within this context, three key concepts can also be considered in this discussion, as they help interpret the temporal and structural dynamics involved in the rising prevalence rates: steepening, no plateau, and resurgence.

- Steepening - The concept of steepening refers to the trend observed in epidemiological curves in which autism diagnoses occur progressively earlier in more recent birth cohorts. This phenomenon suggests that, although the cumulative prevalence of autism remains high, cases are being identified at increasingly younger ages. Several studies indicate that early screening programs and the systematic inclusion of developmental assessments during pediatric check-ups have played a key role in this diagnostic shift [30, 31].

- No Plateau - No Plateau describes the absence of stabilization in prevalence curves over time. Even in older cohorts, new autism diagnoses continue to emerge, which contradicts the expectation that most cases would have been identified in childhood. This trend points to shortcomings in early screening systems and often reflects disparities in access to services, delayed clinical recognition, and cultural differences in the perception of autistic behaviors [32]. Studies of adults diagnosed later in life reveal a distinct profile: a higher proportion of women, individuals with typical intellectual functioning, and a history of adaptive difficulties that went unrecognized during childhood [33]. No plateau also suggests that, despite diagnostic advances, a considerable number of adults remain either undiagnosed or misdiagnosed throughout their lives.

- Resurgence - Resurgence refers to sudden increases in diagnosis rates following institutional, policy, or regulatory changes. A notable example was the publication of the American Academy of Pediatrics (AAP) guidelines in 2007, which recommended universal ASD screening during routine developmental monitoring. U.S. studies report a sharp rise in prevalence curves between 2008 and 2010, particularly among preschool-and school-aged children who previously would not have been systematically screened [30].

These terms, derived from the analysis of longitudinal trends and population cohorts, reveal that changes in prevalence often reflect transformations in the healthcare system, clinical practices, and even the very definition of what is understood as autism, rather than an actual expansion of the condition itself.

## Final considerations

The rise in autism prevalence is a complex and multifactorial phenomenon, influenced by clinical, social, methodological, scientific, technological, and institutional elements. Understanding this landscape requires a multidirectional analysis that integrates multiple layers of interpretation, from advances in biomedical sciences to the sociocultural transformations that shape diagnostic practices.

While diagnostic expansion is evident — and may result in overdiagnosis in certain contexts — a significant number of individuals remain underdiagnosed, particularly among groups such as girls, individuals with typical intellectual functioning, racialized populations, or those from lower socioeconomic backgrounds. These two seemingly opposing phenomena coexist within the same epidemiological field, demanding balanced approaches that are sensitive to the nuances of each case.

In light of this, it is crucial to emphasize the importance of well-trained professionals, with solid technical and theoretical preparation, capable of interpreting diagnostic criteria with both rigor and clinical sensitivity, while also understanding the limitations of early developmental assessments. Diagnoses made by inadequately prepared professionals may lead to inflated statistics, or conversely, may contribute to significant gaps in the recognition of atypical profiles.

Moreover, strengthening epidemiological methodologies—with clearer case definitions, standardized criteria, representative samples, and attention to population heterogeneity — is essential for producing reliable data.

Finally, any analysis of potential overdiagnosis in ASD should not be approached in a reductionist or alarmist manner, but rather grounded in robust evidence, historical context, and a commitment to the well-being of the autistic population. A well-conducted diagnosis is the starting point for access to rights, interventions, and quality of life. However, its trivialization can undermine the credibility of the field, overburden healthcare systems, and divert resources from those who genuinely need specialized support. The challenge, therefore, is not to diagnose more or less, but to diagnose better.

## Conflicts of interest

The authors declare no conflicts of interest.
